# Functional Responses and Prey-Stage Preferences of a Predatory Gall Midge and Two Predacious Mites with Twospotted Spider Mites, *Tetranychus* Urticae, as Host

**DOI:** 10.1673/031.013.0801

**Published:** 2013-01-31

**Authors:** Yingfang Xiao, Lance S. Osborne, Jianjun Chen, Cindy L. McKenzie

**Affiliations:** 1 Department of Entomology and Nematology, Mid-Florida Research and Education Center, University of Florida, Apopka, FL, USA 32703; 2 Department of Environmental Horticulture, Mid-Florida Research and Education Center, University of Florida, Apopka, FL, USA 32703; 3 U.S. Horticultural Reseach Laboratory, USDA-ARS, Fort Pierce, FL, USA 34945

**Keywords:** *Amblyseius swirskii*, *Feltiella acarisuga*, *Neoseiulus californicus*, predation, vegetable crops

## Abstract

The twospotted spider mite, *Tetranychus urticae* Koch (Acari: Tetranychidae), is an important pest of vegetables and other economically important crops. This study evaluated the functional responses and prey-stage preferences of three species of predators, a predatory gall midge, *Feltiella acarisuga* (Vallot) (Diptera: Cecidomyiidae), and two predatory mite species, *Neoseiulus californicus* (McGregor) (Acari: Phytoseiidae) and *Amblyseius swirskii* (AnthiasHenriot), with *T. urticae* as the host, under laboratory conditions. The results showed that *F. acarisuga* was highly effective and the two species of predacious mites were moderately effective in feeding on *T. urticae* eggs. Logistic regression analysis suggested Type II (convex) functional responses for all three species. However, based on the estimates of the handling time and the attacking rates, the three predators had different predation capacities. Among the three species, *F. acarisuga* had the highest predation on *T. urticae*. The maximum daily predation by a larval *F. acarisuga* was 50 eggs/day, followed by a female *N. californicus* (25.6 eggs/day) and a female *A. swirskii* (15.1 eggs/day). A female *N. californicus* produced more eggs than a female *A. swirskii* did when they both fed on *T. urticae* eggs. In addition, all three predator species had no preystage preference for either prey eggs or nymphs. The findings from this study could help select better biological control agents for effective control of *T. urticae* and other pests in vegetable productions.

## Introduction

The twospotted spider mite, *Tetranychus urticae* Koch (Acari: Tetranychidae), is an important pest of vegetables and other economically important crops (Opit et al. 2004; Liburd et al. 2007). Both the adult's and immature's feeding result in leaf abscission, decreased plant vigor, and increased plant death (Fasulo and Denmark 2000). Due to their high reproductive potential and short life cycle, frequent use of miticides has resulted in rapid development of miticide resistance, which has become a major problem for chemical control of this pest (Gorman et al. 2001). In addition, chemical control has been seriously challenged by the current trend towards sustainable and environmentally friendly farming practices (Osborne and Barrett 2005) and by consumers’ demand for safe, fresh vegetables (Dabbert et al. 2004).

Biological control is considered an effective alternative to chemical applications for pest management (Opit et al. 1997). Some reports have documented that several predatory mites were effective in control of *T. urticae* (Rhodes and Liburd 2006; Fraulo and Liburd 2007; Cakmak et al. 2009). However, other reports indicated that the use of some predator species as biological control agents had low efficacy in control of *T. urticae* on vegetable crops (Opit et al. 1997). The low efficacy could be attributed to many factors, including the predator species selected and environmental conditions where the experiments were conducted. To better understand the effectiveness of predator species, a comparative evaluation of optimal predation capability of commonly available predators against *T. urticae* under the same conditions is needed.

The functional response concept first reported by Holling (1959) has been widely used to evaluate the effectiveness of predacious insects and mites (Laing and Osborn 1974; Trexler et al. 1988; Clercq et al. 2000; Reis et al. 2003; Badii et al. 2004; Timms et al. 2008; Xiao and Fadamiro 2010). In general, the functional response of a predator to a prey could be one of the three response models: type I, type II, and type III (Holling 1961; Trexler et al. 1988; Clercq et al. 2000; Timms et al. 2008). In terms of biological control, the most efficient biological control agents exhibited the type II functional response (Pervez and Omkar 2005). Several researchers found that logistic regression analysis is the optimal method in determining the true functional response of a predator to a prey, and is particularly useful in differentiating type II and type III responses by estimation of a linear coefficient (Trexler et al. 1998; Clercq et al. 2000; Timms et al. 2008).

The present study was intended to evaluate the functional responses and prey-stage preferences of *Feltiella acarisuga* (Vallot) (Diptera: Cecidomyiidae), *Amblyseius swirskii* (Anthias-Henriot) (Acari: Phytoseiidae), and *Neoseiulus californicus* (McGregor), with *T. urticae* eggs as the host, in identical laboratory conditions. The selection of the three predator species was based on the following considerations. *F. acarisuga* has been documented as an effective predatory midge for controlling spider mites (Tetranychidae) (Gillespie et al. 1998, 2000; Brodsgaard et al. 1999; Mo and Liu 2007; Xiao et al. 2011). *A. swirskii* is an effective predatory mite currently used for controlling two key pests, *Scirtothrips dorsalis* Hood (Arthurs et al., 2009) and *Bemisia tabaci*, in greenhouses (Lee et al. 2011; Xiao et al. unpublished). However, little is known about whether *A. swirskii* suppressed *T. urticae* when released to control the other pests.. *N californicus* has been used in combination with *Phytoseiulus persimilis* Athias-Henriot for the control of *T. urticae* (Rhodes and Liburd 2006; Fraulo and Liburd 2007; Weintraub and Palevsky 2008; Cakmak et al. 2009). Thus, comparative studies of the predation potential of the three species of predators is a critical first step to select better biological control agents for management of the pests in vegetable production.

The specific objectives of this study were to (1) compare the functional responses of three predator species to *T. urticae* under the same laboratory conditions, and (2) to determine the prey-stage preferences of the three predator species to *T. urticae* under laboratory conditions.

## Materials and Methods

### Host plants

The seeds of the green bean ‘Cangreen’ (Kelloggs Ag. Service., http://www.kelloggseedservice.com/) were sown on Fafard 2-Mix growing medium (Conrad Fafard, Inc., http://www.fafard.com/). The seedlings were transplanted into 8-cm diameter plastic pots filled with Fafard 2-Mix growing medium and enclosed in proof screen cages in air-conditioned rearing rooms or greenhouses (26 ± 2° C, 60 ± 10% RH, 14:10 L:D photoperiod) at the University of Florida's Mid-Florida Research and Education Center in Apopka, FL, USA. The seedlings were pesticide free, and those with uniform size and growth vigor were used for the experiments outlined below.

### Pest mites

Stock colonies of *T. urticae* established originally from multiple locations were maintained on the fully expanded leaves of the green bean seedlings (∼30 days) mentioned above for two years in air-conditioned rearing rooms or greenhouses at the same location. The arthropods and plants in the greenhouses were monitored daily and watered if needed.

### Predatory gall midges

The colonies of *F. acarisuga*, established originally from multiple locations, were maintained on corn mite, *Oligonychus pratensis* (alternative prey), and reared on the corn leaves for 2–3 generations in airconditioned rearing rooms and greenhouses at the location mentioned above. *F. acarisuga* and *O. pratensis-mfested* corn leaves were placed on trays in plastic transparent containers (30 × 40 × 20 cm) with the top holes covered by screen nets for laboratory experimental use. Each tray was isolated by water to prevent mite escape and maintain leaf freshness for up to two weeks.

### Predatory mites

The colonies of *A. swirskii* were purchased from Koppert Biological Systems (http://www.koppert.com/) and reared on the mixed pollen of the plastic trays isolated by water for 2–3 generations before their use for bioassays. The colonies of *N. californicus* were purchased from Biocontrol Network, Inc. (http://www.biconet.com/) and were reared on mixed stages of *T. urticae* on green bean leaves for 2–3 generations before being used for bioassays. All rearing and experiments were conducted under the laboratory conditions mentioned above.

### Functional response of the predatory gall midge

Laboratory experiments were conducted to determine the predation of *F. acarisuga* on *T. urticae* that infested green bean plants based on the protocols described by Opit et al. (1997), Reis et al. (2003), and Xiao and Fadamiro (2010). Bean leaf discs (2.5 cm diameter) were made from same size green bean plants. To obtain different densities of cohorts of eggs for functional response experiments, different numbers of *T. urticae* females (from 2 to 20 per leaf disc) were transferred from the stock colony to leaf discs (one female per leaf disc), allowed to lay eggs for 24 hrs, and then removed from each leaf disc. The number of eggs laid on each leaf disc was counted, and each leaf disc with a range in egg number of 3–5, 6–9, 10–19, 20– 29, 30–39, 40–49, 50–59, 60–69, 70–79, 80– 100, 101–140, and 141–160 was selected and placed on moistened filter paper inside each small Petri dish (3.0 cm diameter × 0.5 cm depth), respectively. One second instar *F. acarisuga* larva was released into each small dish. Four small Petri dishes were fixed in each large Petri dish (15 cm diameter × 1.5 cm) filled with the appropriate amount of water to isolate each small Petri dish. The large Petri dish was sealed with parafilm to prevent arthropod escape and leaf drying. Each density treatment was replicated six times. The controls consisted of discs with the same density of prey eggs without *F. acarisuga*. The number of prey eggs killed by the predator was recorded 48 hrs after the release of *F. acarisuga*.

### Functional response of two species of predatory mites

The functional responses of two phytoseiid species to *T. urticae* eggs were investigated in separate bioassays using a similar procedure as the one described above. The differences were that one gravid female of either *A. swirskii* or *N. californicus* was introduced onto each leaf disc rather than *F. acarisuga*, and the density of 140–160 eggs per arena was not setup in the experiments. The treatments were replicated six times. The number of the prey eggs killed by the predators and the number of eggs laid by each predator (numerical response) were recorded at 48 hrs after the release of either *A. swirskii* or *N. californicus*.

### Types of functional responses and data analysis

In general, the type of the functional response of each predator species was determined by a binomial logistic regression analysis described in detail by several researchers (Clercq et al. 2000, Juliano 2001; Timms et al. 2008; Xiao and Fadamiro 2010), in which the equation *Ne*/*N* = *a* + *bN* + *dN^2^* + *dN^3^* + *e* was used, where *Ne* is the proportion of killed prey; *N* is the number of prey density offered; *a* is the intercept; and *b, c*, and *d* are linear, quadratic, and cubic coefficients, respectively. Type I response was determined by an intercept and constant positive slope (b) > 0. Type II response was the linear coefficient (b) < 0. Type III response was characterized by the linear coefficient (b) > 0 and the quadratic coefficient < 0 (Trexler et al. 1988; Clercq et al. 2000; Timms et al. 2008; Xiao and Fadamiro 2010).

After determining the correct shape of the functional response, a linearization of Holling's disc equation (Holling 1959) based on functional response type was used to estimate parameters (Clercq et al. 2000; Xiao and Fadamiro 2010). The Type II equation used was: *Ne* = *aNT* / (1 + *aNT_h_*), where *Ne* is the number of killed prey, *N* is the number of prey offered, *T* is the total time available for the predator, *a* is the attack rate, and *T_h_* is the handling time. The attack rates and the handling time were estimated to determine differences in the functional responses among the predator species.

The data on the highest (maximum) number of prey eggs killed for each species (70–79 *T. urticae* eggs per arena for *A. swirskii* and *N. californicus*, and 101–140 eggs per arena for *F. acarisuga*) was first normalized by using the square-root (√χ + 0.5) transformation, and then analyzed with one way analysis of variance (ANOVA) followed by TukeyKramer honestly significant difference (HSD) test to determine significant differences in the maximum number of prey consumed among the three species (*p* < 0.05, JMP Version 8.01, SAS Institute Inc. 2009).

### Prey-stage preference experiment

The prey-stage (egg or nymph) preference of each of the three predator species was determined following a similar procedure described by Blackwood et al. (2001) and Xiao and Fadamiro (2010). For each experiment, one second instar *F. acarisuga* larva or an adult female of each of the two predatory mites was confined in a 2.5-cm diameter bean leaf disc arena (as described above). Each leaf disc contained a single ratio of egg to nymph of *T. urticae* with three possible ratios (1 + 0, 1 + 1, 0 + 1) [where (1 + 0) means 40–60 eggs + 0 nymphs, (1 + 1) means 40–60 eggs + 10∼15 nymphs, and (0 + 1) means 0 eggs + 10–15 nymphs], and were tested for prey-stage preference. The experiments were replicated 16 times per ratio for *F. acarisuga* and replicated 12 times for each of the two predatory mite species. The number of each prey-stage eaten by the predator was recorded after 48 hrs.

Prey-stage preference was first calculated with the preference index ß (Manly et al. 1972), as described by Blackwood et al. (2001) and Xiao and Fadamiro (2010).

The *N* (eggs) and *N*' (nymphs) are the number of each prey type provided, and *Nc* (eggs) and *Nc*’ (nymphs) are the numbers of each prey type consumed. This index assigns preference values from 0 to 1, where 0.5 represent no preference. The ß value was calculated for each replicate and averaged to determine the mean ß value for each treatment.

Data on prey-stage preferences was first normalized by using square-root transformation (√*x* + 0.5) and then analyzed with student's *t*-test for each species at two given ratios [(1 + 0) and (1 + 1) or (1 + 1) and (0 + 1) (eggs + nymphs)]. Significant differences between the proportion of each prey stage (eggs versus nymphs) killed were compared by using student's *t*-test (*p* < 0.05, JMP Version 8.01, SAS Institute Inc., 2009).


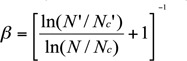


## Results

### Functional responses

**Figure 1.**
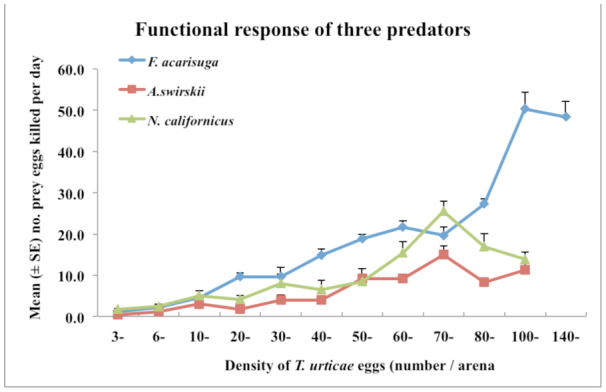
Relationship among the number of *Tetranychus urticae* eggs preyed by larval *Feltiella acarisuga*, female *Amblyseius swirskii*, and *Neoseiulus californicus* with the prey density of *T. urticae* (eggs) provided on bean leaf discs per day. For all three species, the data followed the Type Il convex functional response in which the number of prey consumed increased with prey density up to a maximum point, after which it began to slowly decrease. High quality figures are available online.

For all three species, logistic regression analysis showed that linear coefficient (*b*) was < 0, suggesting that the proportion of prey eggs killed decreased as a function of egg density offered, which was the Type II (convex) functional response (Table 1). The curves of functional responses on *T. urticae* eggs by the three predator species were presented in Figure 1. Natural mortality of prey eggs observed in the control treatment (without any predator) was minimal: 1.5 ± 0.5%, 1.0 ± 0.3%, and 2.6 ± 0.5%. These results suggested that the three predator species effectively predated *T. urticae* eggs.

**Table 1.**
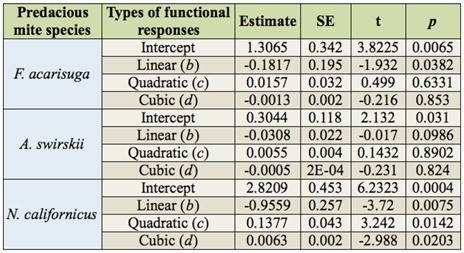
Estimates of coefficients in a binomial logistic regression of the proportion of *Tetranychus urticae* egg eaten by *Feltiella acarisuga, Amblyseius swirskii*, and *Neoseiulus californicus* in relation to total eggs provided on bean leaf discs under laboratory conditions.

**Table 2.**
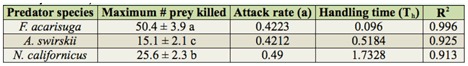
Estimates of functional response parameters from linearization of Holling's Type II model for the three predators *Feltiella acarisuga, Amblyseius swirskii*, and *Neoseiulus californicus* fed *Tetranychus urticae* eggs under laboratory conditions. Means in the second column followed by different letters are significantly different (*p* < 0.05, ANOVA, Tukey HSD).

Comparison of the differences in functional response curves revealed that *F. acarisuga* responded most vigorously at all densities of prey offered, followed by *N. californicus* and then *A. swirskii*. There was significant difference of functional responses between *F. acarisuga* and *A. swirskii* (*F* =*29.46*, df = 1, 110, *p* = 0.0001), between *F. acarisuga* and *N. californicus* (*F* = 9.46, df = 1, 110, *N* = 0.0024), and between *A. swirskii* and *N. californicus* (*F* = 7.32, df = 1,110, *p* = 0.008). Estimates of the parameters [attack rate (a), handling time (Th)] of the functional responses for the three predator species showed that *F. acarisuga* had the shortest handling time, followed by *A. swirskii* and *N californicus*. The three predator species had an almost equal attack rate, suggesting the three predators had similar feeding behavior. This model provided a good fit to data as indicated by high R^2^ values (Table 2).

**Figure 2.**
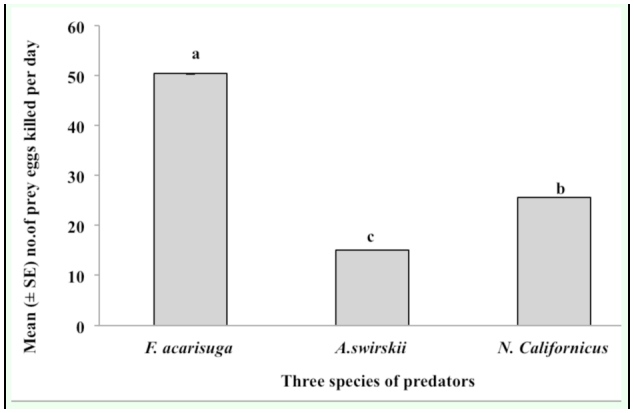
Predation potential of the three predators, larval *Feltiella acarisuga*, female *Amblyseius swirskii*, and *Neoseiulus californicus*, fed *Tetranychus urticae* eggs. Figure shows maximum mean (± SE) number of prey eggs killed by each of the three predators per day at a given density (101– 140 eggs/arena for F. *acarisuga* and 70–79 eggs/arena for two predatory mites). High quality figures are available online.

*F. acarisuga* larvae appeared to be the most effective predator as they consumed a significantly higher number of prey eggs than the other two predatory mite species when comparing the maximum number of prey eggs killed at a prey density (*F* = 30.6, df = 2, 12, *p* = 0.0001). For example, the maximum number of *T. urticae* eggs consumed per day by a larval *F. acarisuga* was 50.4 eggs (∼ 38% predation) at a prey density of 101–140 eggs per arena offered, followed *by a N californicus* female (∼ 25.6 eggs (∼35% predation)) and then *a A. swirskii* female (15.1 eggs (∼ 21% prédation)) at a prey density of 70–79 eggs per arena (Figure 2). However, at lower prey densities (< 19 eggs/leaf disc), the three predator species exhibited similar predation, consuming < 5.0 eggs per disc per day.

**Figure 3.**
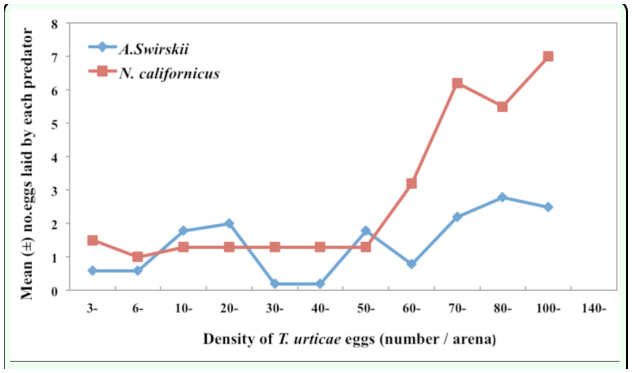
Numerical response: Relationship among the number of eggs laid by female *Neoseiulus californicus* and female *Amblyseius swirskii* with the prey density of *Tetranychus urticae* (eggs) provided per day. Female *N. californicus* showed more eggs laid than femaile *A. swirskii* with the increases of the prey egg densities. High quality figures are available online.

The numerical responses of the two predatory mites (excluding *F. acarisuga* larvae) suggested that a *N. californicus* female produced more eggs than an *A. swirskii* female when they fed on prey eggs (Figure 3). The highest number of eggs laid by *N. californicus* was 8.0 eggs/female within 48 hrs.

### Prey-stage preference

**Figure 4.**
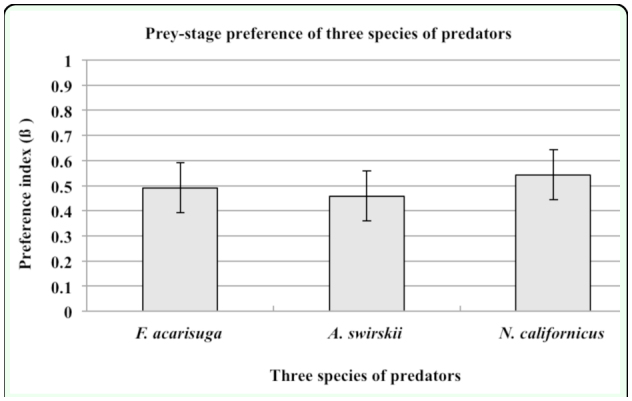
Prey-stage preferences of the three species of predators, larval *Feltiella acarisuga*, female *Amblyseius siwrskii*, and female *Neoseiulus californicus*, when they were provided at a ratio of (1 + 1) eggs to nymphs of *Tetranychus urticae*. Figure shows mean (± SE) preference index (ß). High quality figures are available online.

Based on preference values (β) calculated from above format, the values (β) of *F. acarisuga, A. swirskii*, and *N. californicus* were 0.491, 0.457, and 0.547, respectively, suggesting the three predator species had no prey-stage preference to *T.urticae* stages (Figure 4). All three species fed equally on both eggs and nymphs of *T. urticae* irrespective of prey-ratio when eggs and nymphs were simultaneously provided.

A detailed observation showed that the egg predation rate by *F. acarisuga* was ∼65.7% when only *T. urticae* eggs were offered, the nymph predation rate was ∼62.7% at the presence of only nymphs, and the predation rates of both eggs and nymphs were ∼57.5% when both eggs and nymphs were offered. There was no significant difference in the egg predation rate by *F. acarisuga* between (1+0) and (1 + 1) ratio (eggs + nymphs) (*t* = *1.95*, df = 30; *p* < 0.06), or in the nymph predation rate between (0+1) and (1 + 1) ratio (eggs + nymphs) (*t* = *0.645*, df = 30; *p* < 0.52).

**Table 3.**
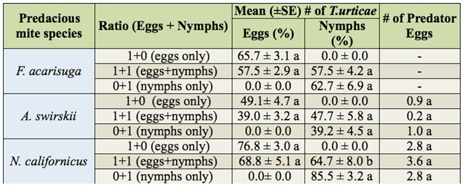
Prey-stage preferences of the three species of predators when different ratios of eggs and/or nymphs of *Tetranychus urticae* offered for 48 hrs under laboratory conditions. For each species, means in the same column followed by same letters are not significantly different (*p* < 0.05, Student's *t*-test). Comparisons were done only for egg predation rate at two ratios ( 1 + 1 vs. 1 + 0) and nymph predation rate at two ratios ( 1 + 1 vs. 0 + 1).

Similarly, for *A. swirskii*, there was no significantly higher egg predation rate when both prey eggs and nymphs were provided compared to when only prey eggs were offered (*t* = *1.77*, df = 22; *p* < 0.08), and no significantly higher nymph predation rate when both eggs and nymphs were provided compared to when only the prey-nymphs were offered (*t* =*1.017*, df = 22; *p* < 0.319). For *N californicus*, there was not a significantly higher egg predation rate when both eggs and nymphs were provided compared to when only eggs were offered (*t* = *1.34*, df =22; *p* < 0.192). However, a significantly higher nymph predation rate was observed when both eggs and nymphs were provided compared to when only nymphs were offered (*t* = *3.178*, df = 22; *p* < 0.004) (Table 3).

## Discussion

### The functional responses of three predator species

The present study showed that the functional responses of the three species followed the Type II (convex) models (Figure 1). This was not surprising since many predators used as successful biological control agents showed the Type II functional responses (Laing and Osborn 1974; Clercq et al. 2000; Reis et al. 2003; Badii et al. 2004; Timms et al. 2008; Xiao and Fadamiro 2010). Opit et al. (1997) reported that *F. acarisuga* exhibited the Type II functional responses to both female and male *T. urticae*, but did not study functional response to *T. urticae* eggs. Our study indicated that *F. acarisuga* larvae also had a Type II functional response to the prey eggs, and confirmed that *N. californicus* had a Type II functional response to *T. urticae* eggs as reported by other researchers (Laing and Osborn 1974; Blackwood et al. 2001; Skirvin and Fenlon 2003; Ahn et al. 2010; Xiao and Fadamiro 2010). There are no other reports of *A. swirskii* showing a Type II functional response to *T. urticae* eggs. The handling time and the attack rate have been used to determine the magnitude of functional responses. The handling time estimate was the cumulative effect of time taken during capturing, killing, and digesting the prey (Holling 1959, 1961). In our study, larval *F. acarisuga* spent the shortest amount of handling time (0.096/hr) to feed on eggs compared to female of *A. swirskii* (0.518/hr) and female of *N. californicus* (1.732/hr), suggesting *F. acarisuga* had a much stronger predation response than the two species of predatory mites. The almost equal attack rates in the three predator species indicated that these predators had similar feeding behaviors (Table 2).

The three predators differed in their percent predation. Larval *F. acarisuga* consumed more prey eggs (> 50 eggs per larva per arena per day at maximum) than female *N. californicus* (25.6 eggs per larva) and female *A. swirskii* (15.1 eggs per larva) (Figure 2). Also, female of *N. californicus* produced more eggs (∼8 eggs per female per leaf disc) than female *A. swirskii* did when fed on the same prey eggs, suggesting that *N. californicus* was more favorable to *T. urticae* than *A. swirskii*. On the other hand, *A. swirskii* is an effective predatory mite currently used for controlling two key pests, *S. dorsalis* (Arthurs et al. 2009) and *B. Tabaci* in greenhouses (Lee et al. 2011; Xiao et al. 2012). Thus, the use of *A. swirskii* could provide an option for controlling multiple pests in vegetable production.

### Prey-stage preferences of the three predators

The results on prey-stage preference suggested that the three predator species equally preferred to feed on both eggs and nymphs of *T urticae*. These findings concurred with reports for *F. acarisuga* (Opit et al. 1997) and for *N californicus* (Blackwood et al. 2001). However, other reports suggested that *N californicus* preferred nymphs over eggs of another mite (Xiao and Fadamiro 2010). The differences may be related to the prey species provided. Comparison of the relative nutritional value of both prey eggs and nymphs revealed that the eggs may be a more profitable prey stage for some predacious mites (McMurty and Rodriguez 1987), suggesting that the predators may prefer to consume mostly eggs in a diet of mixed prey stages.

In conclusion, this study compared the functional responses and prey-stage preferences of three predatory species with *T. urticae* eggs as host under laboratory conditions. The results showed that larval *F. acarisuga*, female *N californicus*, and female *A. swirskii* all exhibited Type II responses to *T. urticae* eggs and had high predation on *T. urticae* eggs, suggesting all three species may be able to effectively regulate populations of *T. urticae* in the field. The results also indicated that the three predators had no preystage preferences to either prey eggs or nymphs. Among the three species of predators, *F. acarisuga* was the most effective predator of *T. urticae*, but *A. swirskii* was a predator of multiple pests. Future studies on interactions of multiple predatory species for managing *T. urticae* and multiple pests are warranted.
